# Physiological, Morphological and Behavioural Responses of Self-Feeding Precocial Chicks Copying with Contrasting Levels of Water Salinity during Development

**DOI:** 10.1371/journal.pone.0165364

**Published:** 2016-10-27

**Authors:** Afonso R. Rocha, Rita Silva, Auxiliadora Villegas, Juan M. Sánchez-Guzmán, Jaime A. Ramos, José A. Masero

**Affiliations:** 1 MARE–Marine and Environmental Sciences Centre, Department of Life Sciences, University of Coimbra, 3000–456, Coimbra, Portugal; 2 Department de Anatomy, Cell Biology and Zoology, University of Extremadura, Avenida de Elvas s/n, 06071, Badajoz, Spain; Universidad de la Republica Uruguay, URUGUAY

## Abstract

Combined physiological and behavioural responses to salt loads during development have rarely been studied in air-breathing vertebrates able to inhabit hypersaline habitats, but they may be of particular importance in understanding, for example, the differences among species in patterns of habitat use or ontogenetic diet switches. Here, we compared the physiological and behavioural responses of self-feeding precocial chicks developed in contrasting levels of water salinity. The model species was the Black-winged Stilt (*Himantopus himantopus*) a precocial shorebird that breeds in a range of habitats from freshwater to hypersaline wetlands. Specifically, we compared resting metabolic rate (RMR), heat shock proteins (Hsp70), plasma ions, hematocrit, body mass, body size, growth rate and head-shaking behaviour of captive-reared Black-winged Stilt fledglings developed under fresh (0 ‰), saline (20 ‰), and hypersaline (60 ‰) water. Contrary to expectations, none of the physiological and morphological variables measured differed significantly among treatments. Likewise, the RMR of wild and captive-reared fledglings was similar. Surprisingly, the saltgland mass of wild fledglings from freshwater and those from hypersaline habitats was also similar. However, head-shaking, a behavioural response associated to minimize salt intake and to expel the secretions of salt glands, differed according to salinity source: head-shaking rate increased with increasing salinity. The results of this study support the key role of behavioural osmoregulation in avoiding salt stress during development.

## Introduction

Salinity is a crucial environmental factor affecting physiological performance in organisms, and represents one of the main natural sources of stress shaping the biodiversity of ecosystems [1]. During their seasonal movements, billions of vertebrates as diverse as fishes, birds, reptiles, marine mammals and amphibians are subjected to large changes in the salinity of their environments. A suite of physiological, anatomical, morphological and behavioural adaptations ensure that they are able to maintain osmotic homeostasis even under highly saline conditions [2–5].

Salinity fluctuations have a significant negative effect on the growth of fishes, amphibians, and marine invertebrates [6–8]. In birds, laboratory and field studies have demonstrated that birds developing under highly saline conditions often exhibit a decreased growth rate, body mass, reduced immune response, and dehydration (reviewed in [5]). However, the life history of a large number of waterbirds shows that they typically rely on saline and hypersaline wetlands as breeding habitats. Many of these waterbirds are precocial species with self-feeding chicks (for example Charadriiformes), so the latter must have a set of co-evolved traits that enable them to deal with saline and hypersaline water. Unfortunately, empirical studies about the potential effects of saline and hypersaline water on self-feeding foraging in precocial chicks are very scarce (but see [9]). As in the case of adult birds, the responses of precocial chicks to salinity should be interpreted in the context of combined physiological and behavioural responses [5,10,11], because the effectiveness of behavioural responses can eliminate the need to invoke comparatively more expensive physiological responses [4,12]. Knowledge of these responses will be critical in understanding the impact of anthropogenically-caused salinization on wildlife, which is a conservation issue of global concern [5,9,13].

The basal metabolic rate (BMR) represents an animal’s maintenance cost, and is useful as a physiological standard for animal performance [14]. In birds, this flexible physiological trait is known to show physiological adaptations to ecological conditions [15,16]. A recent study showed that adult individuals of a shorebird species, the Dunlin (*Calidris alpina*) increased their BMR and daily energy consumption by 17% and 20%, respectively, between freshwater and saltwater [17]. This finding provided strong support that maintaining functional osmoregulatory machinery to excrete excess salt imposes significant energy costs, probably owing to the increased size and metabolic intensity of supraorbital saltglands [17,18], the key organ used by air-breathing animals such as many waterbirds, seabirds and some reptiles to ensure survival in saline environments [5]. It is believed that such costs are unequal in adults and chicks, but studies addressing this question are scarce (reviewed in [5]).

In the case of shorebirds inhabiting hypersaline habitats, precocial chicks of species such as the American Avocet (*Recurvirostra americana*) have relatively large saltglands at hatching [19]. The size of bird saltglands is strongly influenced by salinity levels [16,18,20], so self-feeding chicks of shorebird species breeding across a gradient of environmental salinity could show differences in resting metabolic rate (RMR) related to salinity levels. Studies involving the basal metabolic response of animals during development in relation to environmental salinity will provide new insights into the potential energy costs of inhabiting saline habitats, and the relative flexibility of this physiological trait to deal with salinity.

The ability of some waterbirds to inhabit hypersaline and alkaline wetlands has been suggested to rely on behavioural and, perhaps, anatomical adaptations for reducing the intake of saline water, as well as on the hypo-osmolality of their food supply [11] (see reviews by [5,21]). For example, head-shaking is a behavioural response associated with minimising salt intake and expulsion of salt gland secretions [11,22]. The behavioural and anatomical adaptations are not as well studied as physiological mechanisms, but they may be crucial in maintaining the osmotic balance in these species (reviewed in [5]). The combined study of physiological and behavioural responses of air-breathing animals during development across a gradient of increasing salinity may provide explanations for differences among species in patterns of habitat use, ontogenetic diet switches or in estimating the optimal diet.

**Fig 1:**
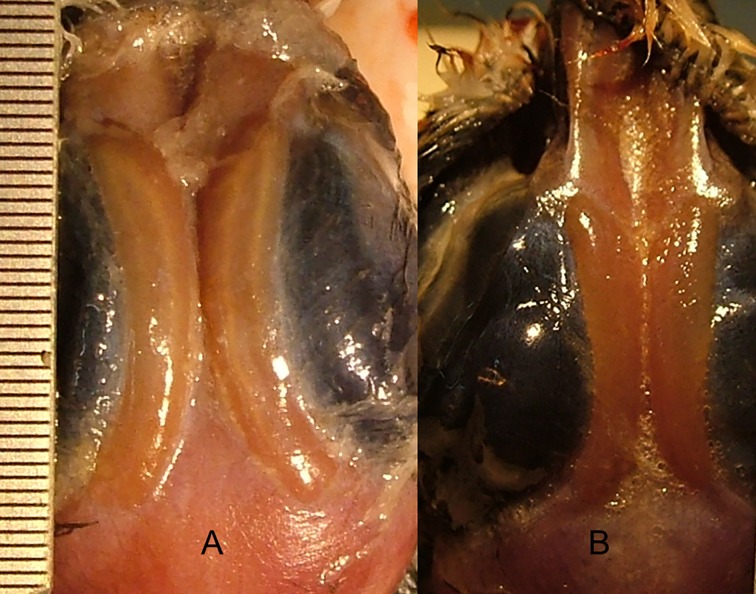
Saltglands. Photographs showing saltglands of Black-winged Stilt wild fledglings found recently dead in freshwater (A) and hypersaline (B) habitats.

Here, we compared the physiological and behavioural responses of an air-breathing precocial species coping with contrasting levels of water salinity during their development. We specifically examined RMR, body mass, growth rate, body size, plasma concentrations of sodium and chloride, hematocrit, heat shock proteins (Hsp), and shaking-behaviour of precocial shorebird chicks developed in freshwater, saline, and hypersaline water. We used the Black-winged Stilt (*Himantopus himantopus*) as a model species (here onwards, stilt), a precocial shorebird that inhabits a variety of habitats during breeding, ranging from inland freshwater, to brackish, saline and hypersaline wetlands [23,24] (Fig 1). This set of physiological variables were measured because they may reflect saline stress in birds [18]. In particular, we measured Hsp70, which is assumed to be the major family of heat shock proteins in vertebrates, and has been used as an index of a range of environmental stressors including salinity (see [17] and references therein).

We predicted that stilt chicks would significantly increase their RMR and head shaking-behaviour with increasing salinity. If stilt chicks ingested large amounts of water and its associated ionic load, we expected that chicks coping with saline and/or hypersaline water during development would show symptoms of saline stress, i.e. higher levels of plasma ions, Hsp70, and hematocrit, and lower body mass, growth rate, and body size than chicks developed in freshwater.

## Materials and Methods

### Captive-Reared Chicks

**Fig 2:**
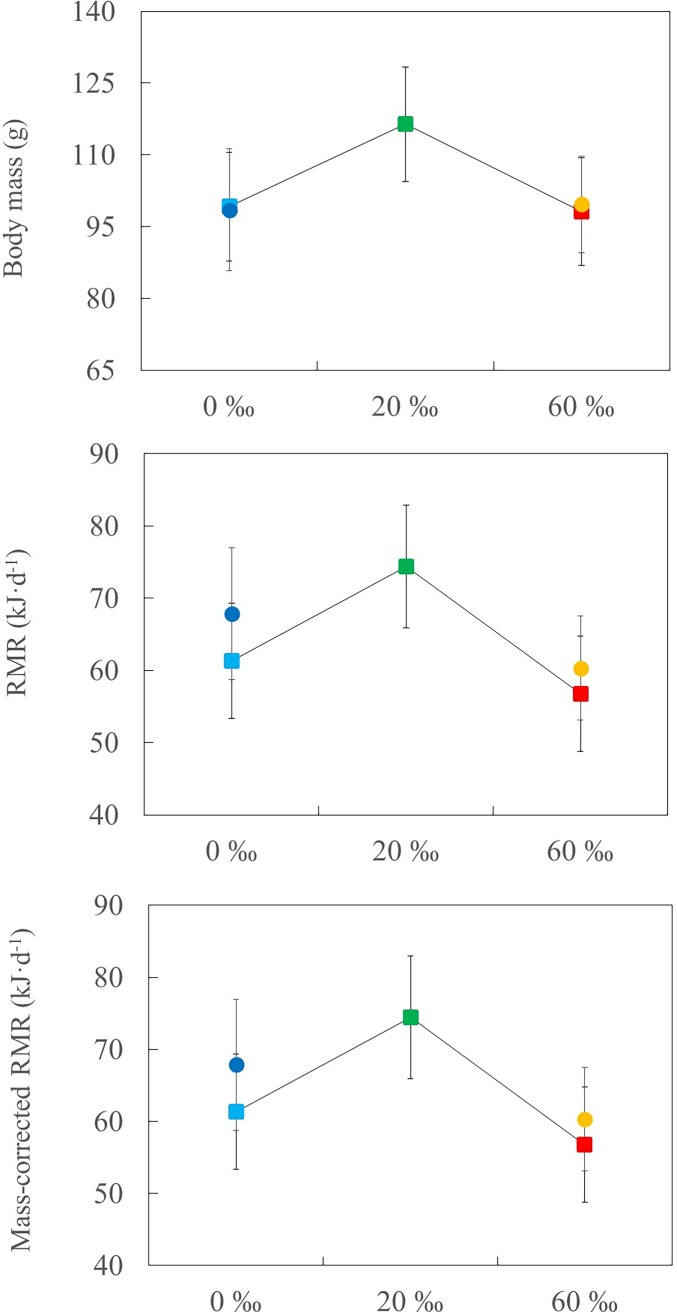
Resting metabolic rate (RMR) and body mass responses in relation to salinity levels. Body mass, whole RMR, and mass-corrected RMR of captive-reared chicks at 0 (n = 9), 20 (n = 8), and 60 ‰ (n = 9) salinity (squares), and wild fledglings from hypersaline pans (n = 8) and freshwater reservoirs (n = 5; circles) (means ± SE). Whole RMR and mass-corrected RMR are presented as least square and adjusted means from the respective ANOVA and ANCOVA models. Data subject to log-transformation are shown as back-log-transformed least-square means. There were no significant differences among treatments (see text for further details).

Wild chicks were captured during spring 2013 in the complex of Samouco saltpans, in the Tagus estuary, Portugal (38°44’N, 8°59’W). The salinity of saltpans ranged from 60 to >300 ‰ during the breeding season (April-July). Here, the stilts use the dykes of the evaporation pans to place their nests [25], so their precocial chicks have to forage on saline and hypersaline water just after hatching. We chose randomly a chick in broods with more than two chicks. Captured chicks were aged according to bill-length (age [days] = -15.67+1.28*bill length [mm]; unpublished data), identified individually with flag codes and transported immediately to outdoor cages (2 × 2 × 2 m) located at the field station of Samouco saltpans, where they were reared in captivity until they fledged. Chicks were randomly assigned to one of the three water salinity regimes, which included the most common salinities through a wide range of distribution: 0 ‰ (n = 9), 20 ‰ (n = 8), and 60 ‰ (n = 9). All cages had available heat lamps, controlled vegetation for shelter, and the same food type. Water was provided in a tray (26 cm diameter, 4 cm high), and its salinity changed according to treatment. The food consisted of live fly larvae (*Protophormia* sp.) and brine shrimp (*Artemia* sp.); both preys were placed into the water tray (Fig 2). Therefore, prey items were always submerged below water surface, so chicks inevitably had to ingest water while feeding (similar to wild conditions). Shorebirds inhabiting hypersaline habitats rely on hypo-osmotic prey such as brine shrimp and diptera larvae and adults, whose body water content is about 78–87% of body mass [11]. The water content of fly larvae provided in our experiment was 73.3 ± 0.5% [17]. The food and water were replaced three times a day, and salinity levels of residual and fresh water were measured using a conductivity meter (HI 98402).

### Morphological and Physiological Measurements

All chicks were weighed (*±* 0.1-precision g) and measured (bill length and tarsus-plus-toe; *±* 0.01-precision mm) daily by the same person (AR) around 14:15 h. For each treatment group, we defined chick growth rate (mm·d^-1^) as the coefficient of a regression of mean body length (tarsus-plus-toe length) on chick age [26].

After three weeks, experimental fledglings were transported in late afternoon to the laboratory of the University of Extremadura for overnight RMR measurements, in terms of oxygen consumption, using standard flow-through respirometry (see full details of the procedure in [16,17]). We performed RMR measurements during the nocturnal period (resting period of the fledglings’ circadian cycle) and in post-absortive state (fledglings were placed in outdoor cages without food for about 3 h but with water *ad libitum* − salinity according to treatment). Fledglings were individually placed in metabolic chambers (15 L) in darkness and housed in a temperature-controlled cabinet at a constant temperature (27°C; within the thermoneutral zone of precocial shorebird chicks [27]). The metabolic chambers received atmospheric air at a rate of 1,000 ml·min^–1^ from calibrated mass flow controllers (MFS-5; Sable Systems, Las Vegas, NV, USA). Water vapour was removed from the air stream immediately downstream from the metabolic chambers using desiccant columns (Drierite®), followed by a multiplexer (TR-RM4; Sable Systems), which allowed automatic switching between four channels. A subsample of the air was taken at 150 ml·min^–1^ using a subsampler mass flow meter unit (SS-3; Sable Systems), and the oxygen concentration was determined using a gas analyzer (FC-10 Oxygen Analyzer; Sable Systems). The latter was calibrated regularly using pure nitrogen and a certified mixture of 21% O_2_ as the low and the high reference, respectively. The oxygen concentration was logged at a 1 Hz sampling rate on a computer using ExpeData software (v. 1.1.25; Sable Systems) and a UI2 converter. Each sampling sequence started with logging ambient baseline air for 10 min, followed by sampling each chamber for 10 min, with the system being flushed for 2 min between samples to remove latent gases. This sequence was repeated four times, so that there were four records per bird per night. Birds were weighed prior to and after RMR measurements, and their mean body mass was used in the analyses (see below). The metabolic rate was calculated using an energy equivalent of 20 J·ml O_2_ [28].

In addition to captive-reared individuals, we also measured RMR in wild fledglings captured in hypersaline (Samouco saltpans; n = 8) and freshwater (reservoirs from Caia, Portugal, 39°00’N, 7°12’W; n = 5) habitats. These birds were captured in the field in late afternoon and then immediately transported to the University of Extremadura for metabolic measurements at night. The wild fledglings from saltpans were captured in hypersaline pans (67 ‰), while wild fledglings from reservoirs only had available freshwater. The procedure for metabolic measurements was identical as described above for captive-reared fledglings.

After RMR measurements, we collected a blood sample (about 70 μl) from the brachial vein using microcapillary tubes. Blood samples were centrifuged for 10 minutes at 11,800 rpm at 4°C to separate plasma from red blood cells, and stored at -20°C until analysis.

Hematocrit was measured as the percentage of blood cell volume to total volume within the microcapillary tube. We used the SPOTCHEM E-Plate with the SPOTCHEM-SE and SPOTCHEM El Analyser, to determine plasma Na^+^ and Cl^-^ concentrations. The SPOTCHEM E-Plate simultaneously measures both electrolytes.

Concentrations of Hsp70 were determined from the erythrocyte lysates by means of an enzyme-linked immunosorbent assay (HSP70 EIA kit; Enzo Life Sciences, Lausen, Switzerland). For details about the heat-shock proteins protocol (see [18]).

After metabolic measurements and blood collection, wild chicks were released at the capture site. Captive-reared chicks were transported again to the outdoor cages at the Samouco saltpans. After one day in these cages, chicks were placed in one outdoor cage (3 × 3 × 1.5 m) located in a pan used by wild stilts as foraging ground. After a few days under these conditions, chicks were released in this pan and they immediately joined the wild flocks.

### Behavioural Response

We videotaped the chicks while they were feeding on fly larvae offered in the water tray. As we described previously, fly larvae were always submerged below water surface, simulating wild feeding conditions (see Fig 2). Therefore, chicks had to cope with the adherent water on each prey item before swallowing it. Each chick was videotaped during the first two minutes of the feeding period, just after renewing the water and food. Thus, for each prey item ingested during the period of recording, we calculated the mean number of head shakes when the prey was handled in the bill, before ingestion, and after ingestion. Each chick was recorded eight times through the growth period.

### Saltglands

We examined the saltglands of fledglings freshly found dead in hypersaline saltpans (n = 14) in the study area and freshwater habitats (n = 6) to assess their dry mass. We performed an incision on birds head scalp with a scalpel to expose salt glands (see Fig 1), and then they were removed, dried to constant weight in an oven (60°C), and weighted in a precision balance to the nearest 0.0001 g.

### Data Analysis

The effect of salinity on fledgling body mass, chick growth rate, body size (tarsus-plus-toe length), plasma concentration of sodium and chloride, hematocrit, Hsp70, and RMR was assessed using general linear models (GLM), with treatment (three or five levels where appropriate) as a fixed factor. To account for the potentially confounding effect of the significant linear relationship between whole RMR and body mass (*F*_1,24_ = 21.50, *P* < 0.001), we also calculated mass-corrected RMR by using an ANCOVA with body mass as a covariate. A General Linear Mixed Model (GLMM) was used to test the effect of salinity in the rate of head-shaking, with treatment as a fixed factor (three levels) and individual identity as a random factor to avoid the lack of independence in the observations (note that each chick was videotaped several times; see above). Morphological and physiological differences between wild and captive-reared fledglings according to salinity were tested using *t*-test for independent samples, except in saltgland mass for which we used a GLM with size (tarsus-plus-toe length) as covariate. To assess the robustness of the analyses, a post-hoc statistical power (1-*β*) was calculated by using G*Power 3.1. RMR, body mass, tarsus-plus-toe length, plasma concentration of ions and lateral head-shakes were log transformed to achieve normality and homogeneity of variances. Data are shown as mean ± SE. All statistical analyses were performed with STATISTICA v10.

### Ethics Statement

The experiments described in this study were performed in the field station of Samouco saltpans, a private company of salt production. They were carried out in accordance with the Institute of Nature Conservation and Forestry guidelines (the State entity with responsibility for approving these experiments and bird captures), with permission number 295 E 296 /2013/CAPT. The experimental protocol was approved by the bioethical committee of the University of Extremadura, with permission number 24//2011.

## Results

**Fig 3:**
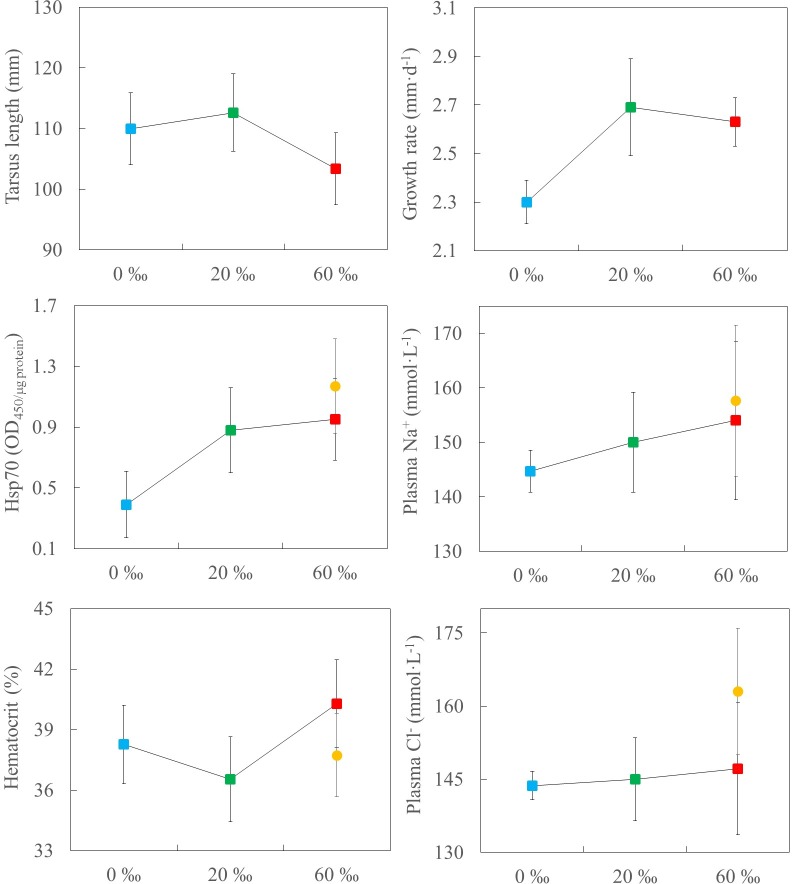
Morphological and physiological responses. Measurements of Black-winged Stilt fledglings grown at 0, 20, and 60 ‰ salinity (squares). These measurements are also reference values for wild fledglings from hypersaline pans (circles). Data are means ± SE (data subject to log-transformation are shown as back-log-transformed least-square means). There were no significant differences among treatments (see text for details).

Prior to the experiment, body mass and body size (tarsus-plus-toe length) of chicks were similar among treatments (*F*_2,23_ = 2.54, *P* = 0.10, *F*_2,23_ = 1.87, *P* = 0.18, respectively; 1-*β*: 1.00, in both cases). The age at capture differed to some extent among captive-reared chicks, but there was no significant differences in the mean number of days on each treatment (*F*_2,23_ = 1.48, *P* = 0.25, 1-*β*: 0.99). We did not find significant effects of treatment in body mass, whole RMR, and mass-corrected RMR of fledglings (Table 1, Fig 2). We did not find significant differences among treatments in growth rate, body size, plasma ion concentrations, hematocrit, and Hsp70 levels of fledglings (Table 1, Fig 3).

**Table 1:** Salinity treatment effects on the physiological and behavioural responses of stilt chicks reared in captivity. Statistically significant differences are shown in bold. Statistical power (1-*β*) was calculated for each variable. See text for details.

	*F*	*P*	*1*-*β*
Body mass	*F*_2,23_ = 0.61	0.55	0.74
Growth rate	*F*_2,18_ = 1.47	0.26	0.99
Body size	*F*_2,23_ = 0.50	0.61	0.61
Whole RMR	*F*_2,23_ = 1.21	0.32	0.99
Mass-corrected RMR	*F*_2,22_ = 0.57	0.57	0.68
Plasma Na^+^	*F*_2,15_ = 0.24	0.79	0.16
Plasma Cl^-^	*F*_2,15_ = 0.01	0.99	0.05
Hematocrit	*F*_2,15_ = 0.99	0.39	0.99
Hsp70 levels	*F*_2,15_ = 1.69	0.22	1.00
Head-shake movements*	*F*_2,23_ = 30.34	**< 0.001**	1.00
Head-shake movements**	*F*_2,23_ = 13.04	**< 0.001**	1.00

*before prey ingestion

**after prey ingestion

When we included all five chick groups in the analysis, whole RMR (*F*_4,34_ = 0.98, *P* = 0.43, 1-*β*: 0.99), mass-corrected RMR (*F*_4,33_ = 0.62, *P* = 0.65, 1-*β*: 0.83), and body mass (*F*_4,34_ = 0.98, *P* = 0.45, 1-*β*: 0.88) did not differ among them (see mean values in Fig 2). Unfortunately, some blood samples of wild freshwater fledglings were unfrozen accidentally before analyses and were discarded from further analyses. Due to the low size sample, we only compared plasma ions, hematocrit, and Hsp70 between wild fledglings from hypersaline habitats and those captive-reared at 60 ‰; there were no significant differences between both groups (Na^+^: *t*_12_ = 1.25, *P* = 0.24; Cl^-^: *t*_12_ = 0.53, *P* = 0.61; Hematocrit: *t*_12_ = 0.52, *P* = 0.61; Hsp70: *t*_11_ = -0.48, *P* = 0.64; see mean values in Fig 3).

**Fig 4:**
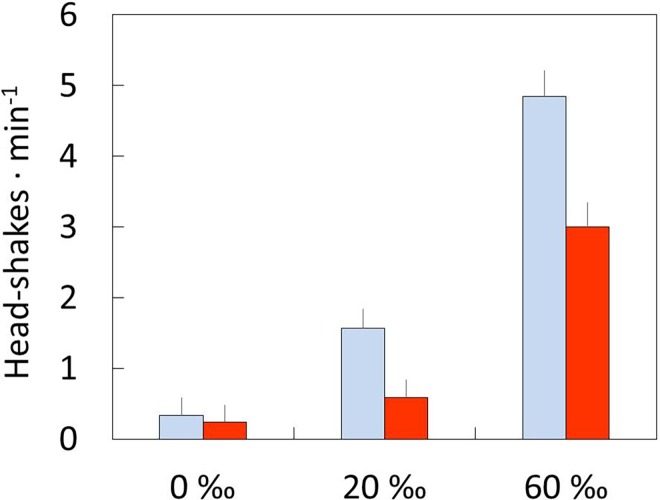
Behaviour response. Head-shaking behaviour (mean ± SE) of captive-reared stilt chicks before (grey bars) and after (white bars) ingesting diptera larvae at 0, 20, and 60 ‰ salinity. There were significant differences among treatments (see text for further details).

The saltgland dry mass was similar in wild chicks from hypersaline pans (43.0 ± 12.3 mg) and freshwater reservoirs (55.0 ± 10.8 mg; *F*_1,6_ = 0.48, *P* = 0.51, 1-*β*: 0.35). Lastly, head-shaking behaviour differed significantly among treatments, with chicks from hypersaline water showing the highest rate of head-shake movements (Table 1, Fig 4). Post-hoc Tukey test showed significant differences among all treatments (*P* < 0.001), except between treatments 0 and 20 ‰ after prey ingestion (*P* = 0.11).

## Discussion

It has been shown recently in adult shorebirds that maintaining active osmoregulatory machinery is energetically expensive [reviewed in 5]. However, this study supports that chicks reared at 20 or 60 ‰ salinity did not face major osmoregulatory costs, based on the fact that there were no significant differences in RMR, growth rate, body size, and body mass among the salinity treatments. The absence of differences in haematocrit, Hsp70 and plasma osmoregulatory parameters among the salinity treatments also suggested that chicks are able to successfully maintain their ionic and osmotic homeostasis in challenging saline and hypersaline habitats. Nevertheless, the lack of significant differences in plasma osmoregulatory parameters must be interpreted with caution owing to the low power of the statistical analysis. This study showed that salinity affected head-shaking behaviour, highlighting the significant role of behavioural responses in the avoidance of salt-loading by self-feeding precocial offspring in species such as Black-winged Stilt.

### Saltglands

Maintaining and using large, active saltglands is assumed to impose significant energetic and physiological costs, and adult waterbirds adjust the mass of saltglands to changing osmoregulatory demands (reviewed by [5,18]). As noted earlier, a recent study by [17] found that BMR and daily energy consumption of adult Dunlins increased significantly between freshwater and seawater, suggesting that developing and maintaining active saltglands is energetically expensive. In contrast to our prediction, whole and mass-corrected RMR of fledglings were similar among treatments, which suggested that the energetic costs of maintaining an active osmoregulatory machinery were low or masked by the costs of growth. The RMR values from wild fledglings corroborated that the captive-reared fledglings’ RMR were within natural ranges. We expected that the saltglands of fledglings from hypersaline habitats should have the highest mass, but hypersaline and freshwater fledglings had similar saltgland mass. Although the latter should be interpreted with caution due to the low sample size and statistical power, the lack of an effect of salinity on metabolic cost suggests that these energetically expensive glands from hypersaline and freshwater stilt chicks were similar in mass and/or function.

The degree of hypertrophy of salt glands in adult shorebirds is strongly influenced by salinity levels, but also by factors such as ambient temperature, prey type, and energy intake rates [16]. However, almost all data on saltgland mass of shorebirds is based on studies performed with adult individuals. In the case of American avocet, a related shorebird species which also uses habitats across a very wide range of salinities, it has been shown that their chicks hatched with saltglands that were relatively large, similar to those of adults or to those of some marine birds [19]. It has been argued that there is an endogenous rhythmicity in the size and activity of adult waterbird saltglands (reviewed in [5]). If stilts also hatch with large saltglands, and their size and activity are under endogenous control, independent of the environmental salinity, it could explain why this extra-renal salt-secreting structure showed similar mass across habitats.

### Morphological and Physiological Responses

We found no evidence for salt stress, since growth rate, body size, body mass, hematocrit, plasma concentration of sodium and chlorine ions, and Hsp70 levels of stilt chicks did not vary according to treatment. These data suggest that stilt chicks ingest very little water while feeding, at least when they feed on saline and hypersaline water.

The head-shaking behaviour of stilt’s chicks increased significantly with increasing salinity, suggesting that this behaviour was highly effective in reducing the intake of ions that would have to be excreted otherwise. Adult shorebirds such as American Avocets and Wilson’s Phalaropes (*Phalaropus tricolor*) as well as other waterbird species such as Eared Grebes (*Podiceps nigricollis*) rely strongly on behavioural adjustments and anatomical adaptations of the feeding apparatus (bill, tongue, and oral cavity) to avoid ingesting large amounts of saline water [10,11]. In our model species, bill morphology and oral cavity can play a significant role in reducing this intake of ions. Many shorebird species, including stilts, phalaropes and small-sized calidrids, possess needle-shaped bills, which allow them to pick prey cleanly from the water column [11,29,30], to spurt water from their bills when feeding in shallow water [31], and to use ‘surface tension transport’, a feeding mechanism employing the surface tension of water surrounding prey to transport prey in millimetric droplets from bill tip to mouth [29,30,32]. These feeding mechanisms could minimize salt ingestion, as they include the disposal of the transported salt water [33,34]. The morphology of the oral cavity, with palatal papillae, also could promote lateral water drainage (see details in [11]). In contrast, other species such as Red Knots (*Calidris canutus*) which have relatively large saltglands and do not feed extensively on brine shrimp probably because their thick bills do not enable them to feed on this prey without consuming hypersaline water [33].

### Hypo-Osmotic Prey

The growth, behaviour and survival of captive-reared chicks of the American Avocet were examined in relation to a range of salinities (freshwater, brackish, saline, and hypersaline water) [9]. Although they found that hypersaline-reared chicks significantly increased their head-shaking behaviour, they also reported negative effects of increasing salinity, resulting in a significant body mass loss and dehydration of avocet chicks. In our experiment, chicks fed on hypo-osmotic prey items with a large amount of body water (73%). It is important to note that in the study by [9] provided food consisted of an artificial mixture of small-grain trout chow (pelleted food), egg yolk, coarsely ground oyster shell, and vitamin and mineral supplements. The water content of this food type was most likely significantly lower than the body water of natural hypo-osmotic prey found in hypersaline habitats, since the body water content of these prey items from hypersaline habitats is >78% [11]. In hypersaline habitats, hypo-osmotic invertebrate prey probably provide most or all of the water that shorebirds need [11]. The contrasting results of the study by [9] and those found in our experiment suggest the importance of the availability of hypo-osmotic prey with high body water content for waterbirds inhabiting hypersaline habitats.

Obviously, the fact that shorebird chicks cope successfully with saline and hypersaline water is not surprising, given the large number of waterbirds that use natural hypersaline lakes as breeding habitats. Today, a large number of shorebirds and other waterbird groups rely on saltpans ‒ hypersaline anthropogenic habitats used to obtain salt by seawater evaporation ‒ for breeding [25]. Previous findings [9] could question the suitability of this and other hypersaline habitats for the development of precocial chicks of several shorebirds such as Kentish (*Charadrius alexandrinus*) and Snowy (*Charadrius nivosus*) plovers, Avocets or Black-winged Stilts, but we showed that these precocial chicks could grow successfully in hypersaline habitats when hypo-osmotic prey is available.
